# Illuminating consciousness

**DOI:** 10.3389/fpsyg.2026.1751131

**Published:** 2026-05-08

**Authors:** Conor H. Murray, Kafui Dzirasa, Dana Sawyer, Karen Waconda-Lewis, Landon Pollack, Louis J. Muglia

**Affiliations:** 1 Department of Psychiatry and Biobehavioral Sciences, David Geffen School of Medicine, Jane and Terry Semel Institute for Neuroscience and Human Behavior, University of California, Los Angeles, Los Angeles, CA, United States; 2 Howard Hughes Medical Institute, Chevy Chase, MD, United States; 3 Department of Psychiatry and Behavioral Sciences, Neurobiology, and Neurosurgery, Duke University Medical Center, Durham, NC, United States; 4 Department of Biomedical Engineering, Duke University, Durham, NC, United States; 5 Maine College of Art & Design, Portland, ME, United States; 6 Center for Native American Integrative Healing, LLC, Tijeras, NM, United States; 7 School of Medicine, Family and Community Health, University of New Mexico, Albuquerque, NM, United States; 8 Harbor-UCLA Medical Center, Torrance, CA, United States; 9 Burroughs Wellcome Fund, Durham, NC, United States

**Keywords:** consciousness, neurophenomenology, ontology, philosophy of mind, structuralism

## Abstract

What consciousness is, and how it relates to the body and nature at large, are among the most enduring questions in human history. Notable thinkers have long grappled with its definition, mechanisms, and purpose. In this review, we examine both historical and contemporary perspectives on consciousness across philosophy, science, medicine, and practice. By integrating diverse perspectives and lines of evidence, from neuroscientific models to clinical applications and contemplative methods, we aim to synthesize insights into a more holistic framework of consciousness. Our objective is two-fold: first, to identify common themes and persistent gaps in knowledge, and second, to highlight critical opportunities for future investigation. In doing so, we advance a working model of consciousness that considers how consciousness is constructed, how it can be measured and modified, and why it may be central not only to survival, but also to human flourishing.

## Introduction

What is consciousness? In one sense, consciousness is the one thing all of us are more certain of than anything else, the one thing that is the most proximal to us. And in another sense, we have a better scientific understanding and explanation for those things that are most distal to us, from distant galaxies to quantum particles. The word consciousness itself can be a source of confusion, as the word can represent a number of related, but distinct concepts. For instance, consciousness may refer simply to wakefulness or arousal, or more precisely, to the phenomenal, inner first-person experience of subjectivity, or to the access of information and content, or to the awareness of self or of space and time, or the various states that may alter normal waking consciousness along or beyond these fundamental dimensions. Toward illuminating consciousness, we are primarily concerned with how consciousness has been described and operationalized from diverse vantage points to gain a comprehensive overview that uncovers persistent gaps in knowledge and points to new horizons for future investigation. Where appropriate, we will contextualize how consciousness is specifically defined or operationalized according to the perspectives of the authors, scientists, medical doctors, and practitioners we convey. From a holistic vantage point, our scoping review provides a high-level overview across the domains of philosophy, science, medicine and practice and highlights critical opportunities for future investigation while pointing to a novel working model of consciousness.

## Philosophical foundations

Questions about the nature of the mind, of consciousness, are as old as philosophy itself, arising across cultures throughout human history. Contemporary philosophical perspectives, from which today’s scientific study of consciousness is situated, are shaped by rich and extensive philosophical works that have emerged across the globe over time.

### Ancient India

The oldest philosophical texts known to place an emphasis on consciousness are the *Upanishads* (800–200 BCE), where consciousness – referred to as *cit –* is a fundamental nature of reality. The texts point to being, consciousness, and bliss (*sat-cit-ananda*) as three ways of describing *brahman*, the singular, infinite, and ultimate reality underlying all appearances. The individual self, stripped of all physicality and sensation as pure witnessing awareness, is referred to *atman*. Central to Upanishadic philosophy is that *atman* and *brahman* are actually identical – the individual witnessing self, when fully understood, is not a fragment, but a reflection of ultimate reality, with *cit* operating simultaneously at both levels, revealing the non-dual nature of universe and self. One’s level of consciousness at the *brahman,* universal, or cosmic scale is known as *samadhi* in Hinduism and *rigpa* in Tibetan Buddhism. While these terms are concerned with the metaphysical or ontological orientation of consciousness within the fundamental nature of reality, Upanishadic philosophy also described consciousness in terms of v*ijñāna,* that is consciousness of something, the familiar consciousness that we have of all things in our awareness at each passing moment (Deutsch, 2004).

### Ancient China

In China, Daoist philosophy associated with Laozi (sixth century BCE) and Zhuangzi (369–286 BCE), similarly described non-dual states of consciousness, where conventional boundaries, including between self and world, dissolve into an undifferentiated and natural flow of reality, the *Dao*. The state of mind with perfect alignment with this natural flow is described as *wu wei*. Confucianism, which developed alongside Daoism, emphasized moral awareness, arguing for applications of consciousness in the form of compassion and discernment (Ziporyn, 2020).

### Ancient Greece

The father of Western philosophy, Socrates (470–399 BCE), viewed the *psyche* as the seat of intelligence and morality, and that care for the *psyche* through virtue and knowledge was the greatest human endeavor. While Plato (428–348 BCE) argued the *psyche* is of a non-material nature, with knowledge gained through mental insight rather than physical sensation, Aristotle (384–322 BCE) disagreed, arguing that the *psyche* is the essential material form (*morphe*) of a living body. This disagreement concerning whether consciousness is fundamentally distinct from or intrinsic to the body persists as a central tension in contemporary philosophical perspectives today known as the mind–body problem (Caston, 2006).

### Medieval Middle East

Prior to the beginnings of modern philosophy, Ibn Sina (980–1037) proposed the “Floating Man” thought experiment, arguing that a man fully suspended in darkness, deprived of all sensory input would nonetheless be aware of his own immediate existence. This thinking self, without any reference to the body whatsoever, supported the notion that consciousness is distinct from the body. Al-Ghazali (1058–1111) inquired further about how to gain knowledge about the mind, arguing that consciousness can only be understood through the direct, first-person perspectives, as opposed to any third-person perspective or rational argument (Sina and Rahman, 1952).

### Early modern period

René Descartes (1596–1,650), considered the father of modern philosophy, formalized the mind–body problem in what is known today as dualism (Priest, 1981). Like the “floating man,” he argued that the mind and body are two distinct substances: a thinking substance (*res cogitans*), which clearly and distinctly demonstrates the proof of one’s existence (*cogito, ergo sum*), versus an extended, bodily substance (*res extensa*) that is vulnerable to deception by the senses. While Descartes addressed the distinction between the mind and body as separate substances, he left open the question as to the nature of their interaction. Baruch Spinoza (1632–1677) responded by proposing that the mind and body are not two substances but two attributes of a single underlying reality, today known as dual-aspect monism (Solms, 2019). Immanuel Kant (1724–1804) later emphasized that our senses can never know the true nature of things in the world (*noumena*) and are limited only to how things appear to us through our senses (*phenomena*). Kant’s work established a foundation for phenomenology, arguing that experience has a structure that is organized by categories like time, space, and causality, but stopped short of systematically examining these phenomenological structures.

### Nineteenth and twentieth centuries

Georg Wilhelm Friedrich Hegel (1770–1831) advanced phenomenology through deep introspection. From insights gained through direct observations of his mind, he argued that consciousness is a dynamic process that unfolds dialectically, a process involving contradictions and their resolution, opposites becoming equal, over time. Edmund Husserl (1859–1938) later established phenomenology as a rigorous first-person science of consciousness, one where assumptions about the external world should be suspended (*epoché*) to examine the pure structures of experience. Husserl rejected mind–body dualism, arguing that the world is constituted within consciousness, a form of monism known as idealism (Kelly et al., 2015). Jean-Paul Sartre (1905–1980) later adapted phenomenology for his own existentialist philosophy. Phenomenology also informed the science of neurophenomenology, described in detail below, as well as heterophenomenology, a term introduced by Daniel Dennett (1942–2024), describing third-person methods to gain insight into what people say about their experiences, while suspending assumptions about the veracity of first-person experience entirely (Dennett, 1991, 2003).

Nineteenth and twentieth century philosophers also emphasized the role of logic and language for clarifying philosophical questions, including those related to philosophy of mind. These individuals included Gottlob Frege (1848–1925), Alfred Whitehead (1861–1947), Bertrand Russell (1872–1970), and Ludwig Wittgenstein (1889–1951). In the latter twentieth century, philosophers developed notable thought experiments to support claims about consciousness, including Saul Kripke (1940–2022), who challenged the dominant scientific and physicalist view (that consciousness is explainable in terms of physical processes in the brain) by conceiving of the experience of pain without any corresponding neural event. More recently, John Searle (b. 1932), through the “Chinese Room” thought experiment, argued that an artificial intelligence system should be no more capable of understanding a conversation in Chinese than a person locked in a room tasked with manipulating Chinese symbols to produce appropriate outputs, despite lacking comprehension of the Chinese language. In other words, syntactic processing alone, however sophisticated, is insufficient to generate genuine understanding or subjective experience.

### Toward a science of consciousness

Over millennia, philosophical inquiry has converged on a set of questions that modern empirical research seeks to address: What is the relationship between the mental and the physical? Is consciousness a product of complex physical organization, or something more fundamental? Can first-person experience be reliably reported and rigorously studied? In the search to explain how subjective experience exists in terms of physical or neural processes, aptly named the “hard problem of consciousness” (Chalmers, 1995), four broad ontological positions emerge: physicalism, idealism, dualism, and dual-aspect monism, which continue to frame the science, medicine, and practice of consciousness described in the proceeding sections below.

## The science of consciousness

The science of consciousness aims to model and test falsifiable hypotheses to better understand the place of consciousness in the natural world. Through rigorous data collection, investigators are now mapping and uncovering important clues to the nature of mind. In this section, we review the current state of the science of consciousness including descriptions of modern methods and theories of consciousness. Here, consciousness primarily refers to the experience of wakefulness or recognition of stimuli and its associated neural correlates, including specific distinctions associated with various methodologies and theoretical considerations.

### Consciousness in life on Earth

**Figure 1:**
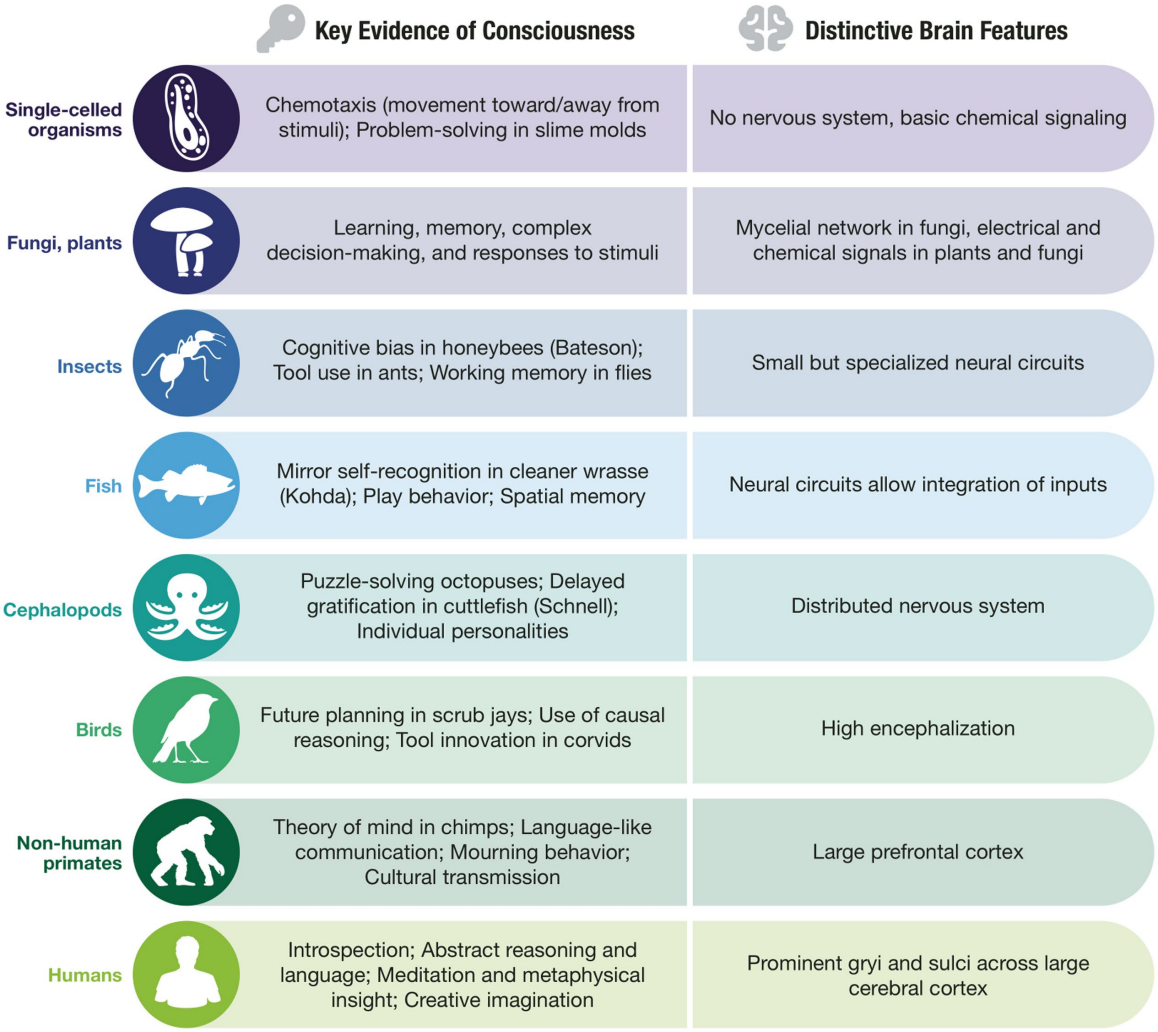
Consciousness in life on Earth. Here, we demonstrate levels of consciousness (in varieties of perception and cognition) across species from single-celled organisms to humans, who possess the most complex natural object known in the universe, the human brain.

As whales sleep, they put one hemisphere of the brain asleep at a time (Lyamin et al., 2002). What is it like to sleep this way? For Descartes, animals or “automata” were denied a conscious mind, incapable even of experiencing pain. Modern thinking on consciousness for animals and other organisms is shaped by Thomas Nagel’s 1974 essay “What Is It Like to Be a Bat?,” which argues that an organism has consciousness if there is something it is like to be that organism (Nagel, 1980). Many conscious experiences may be conserved across animal species, as consciousness appears to provide a biological solution for integrating information about self and world. Researchers have reported behaviors in animals that indeed suggest meaningful internal states. Several examples include: cognitive biases in honeybees (Bateson et al., 2011), delayed gratification in cephalopods (Ponte et al., 2022), and mirror self-recognition in fish (Kohda et al., 2019). Today, researchers call for greater scientific evaluations of animals, plants, and fungi to examine the diversity of consciousness across species (Andrews et al., 2025; Brown et al., 2024; Calvo et al., 2019) (Figure 1). These and other studies, including even in single-celled organisms (Reber et al., 2023), indicate roles for consciousness that challenge traditional boundaries.

### Methods in consciousness research

The first formal laboratory of psychology was built by Wilhelm Wundt (1832–1920). In *An Introduction to Psychology*, Wundt wrote: “This science has to investigate the facts of consciousness, its combinations and relations, so that it may ultimately discover the laws which govern these relations and combinations” (Wundt and Pintner, 1912). However, his use of introspection, or “internal perception” lacked tools for observing, reproducing, or experimentally modulating internal perceptions, ultimately allowing room for behaviorism–the study of human and animal behavior–to dominate the field of psychology.

In the mid-20th century, experimental psychology began to warm to internal states in a “cognitive revolution.” Toward the end of the century, modern neuroscientific methods for investigating neural correlates of mental states and consciousness became available in hospital and research systems. These neuroimaging approaches include functional magnetic resonance imaging (fMRI), with the spatial resolution to identify regional activations linking neural networks, and electroencephalography (EEG) with the temporal resolution to examine evoked responses, oscillations, and asynchronous neurophysiological activity in the brain. The field of neuroscience that emerged sought to gain insight into the mind indirectly, treating the brain, and not consciousness, as a starting point.

By the end of the 20th century, Francisco Varela (1946–2001) became a prominent champion of a new method that sought to combine the rigor of first-person phenomenology with the rigor of third-person neuroscience, an approach known as neurophenomenology (Varela, 1996). Over the past two decades, empirical studies have begun to realize Varela’s vision. For instance, recent efforts in micro-phenomenology have paired discrete moments of experience with specific neural correlates, informing the neural substrates of diverse human experiences (Petitmengin et al., 2019).

Today, neuroscientists are mapping the brain in search for the neural correlates of consciousness (NCCs), the minimal neural mechanisms jointly sufficient for any conscious experience (Chalmers, 2000; Crick and Koch, 1990; Koch et al., 2016; Lepauvre and Melloni, 2021). Combining neuroimaging and brain mapping techniques with behavioral paradigms, researchers have begun to illuminate the neural architectures of consciousness, pointing to a role for recurrent, or feedback, processing in supporting conscious awareness (Box 1). In these paradigms, consciousness is often operationalized through participants’ self-report. One critique is that a behavioral paradigm may conflate the “what it is like,” known as phenomenal consciousness, with what is merely accessible for the report, known as access consciousness (Block, 1995). To address this, no-report paradigms have replaced explicit reports with implicit indicators, such as pupil dilation. Findings from no-report paradigms typically correlate conscious awareness with posterior cortical areas (Koch et al., 2016), whereas report-based paradigms typically correlate with frontal brain activity. However, no-report paradigms may not sufficiently address all confounding variables. In the absence of explicit reports, post-perceptual cognition likely remains in play to obscure results on NCCs (Block, 2019). Further, any NCC that could be detected through report or no-report paradigms should be considered a “content-specific NCC,” representing the specific content imposed by the task design, such as a visual stimulus presentation of a face, as opposed to a “full NCC,” or mechanism that is jointly sufficient for being conscious, irrespective of particular contents (Koch et al., 2016).

Box 1: **Behavioral Paradigms in Consciousness Studies**

*Binocular rivalry*

When the eyes are presented with incompatible patterns, only one pattern is “conscious” at a time, as operationalized by participant self-report, with frequent alternations reported between the two patterns (Wheatstone, 1838). This binocular rivalry provides a paradigm for uncovering competing processes of visual awareness in the brain (Blake et al., 2014). Recent work has localized the rivalry in the thalamus, indicating the conscious rivalry is engaged early within the visual processing hierarchy (Yildirim and Schneider, 2023).

*Visual masking*

When two stimuli are presented in very rapid succession (typically within 50 ms), the second masks the first from access to self-report. Nonetheless, “unconscious perception” of the first stimulus influences subsequent behavior (Ludwig, 2023). Visual masking has revealed that conscious perception requires feedback or “recurrent processing” in the brain, where the sensory information from visual cortices must be relayed back to visual cortex via higher-level associative cortices for self-reported awareness (Fahrenfort et al., 2007).

*Attentional blink*

On a slower timescale, when a target stimulus is presented 200–500 ms after an initial stimulus, the target is often not detected (Kranczioch et al., 2005). EEG detects event-related potentials (ERPs), neural responses time-locked to specific mental events. A positive deflection 300 milliseconds after an event, known as the P300, is associated with the recognition and evaluation of a stimulus. During the attentional blink paradigm, P300 amplitudes are reduced, or even absent, following the second target, reflecting the failure to detect (Kranczioch et al., 2007). However, outside of the attentional blink paradigm, the P300 lacks empirical support for being a neural correlate of consciousness, with P300 ERPs evoked in the absence of conscious awareness (Melloni et al., 2011; Silverstein et al., 2015).

*Change blindness*

When differences in a scene are not detected, this is referred to as change blindness. Both fMRI and EEG have been used to observe markers of this detection in the brain. For fMRI, this includes the activation of a network involving frontal and parietal cortices, in addition to subcortical regions including a nucleus of the thalamus (Beck et al., 2001; Pessoa and Ungerleider, 2004). Using EEG, researchers have identified larger amplitudes of ERPs during trials when participants detect changes, compared to when they do not, similar to findings in the attentional blink paradigm (Scrivener, 2020).

### Theories of consciousness

Debates for and against theories of consciousness continue alongside calls toward unifying frameworks. Here, we discuss several contemporary theories (Box 2).

Box 2: **Theories of Consciousness**

Integrated Information Theory

*Epistemological status: empirically grounded; some claims contested as unfalsifiable*

Integrated Information Theory (IIT) begins with consciousness, not the brain, with the premise that experience is a unified integration of various cognitions and perceptions (Tononi, 2004). IIT argues that consciousness is irreducible to discrete components and that the level of integration is quantifiable and identical to the extent that a system has consciousness (Oizumi et al., 2014). IIT is a mathematical model of consciousness that has also been described as a pseudoscience (Lenharo, 2023). Nonetheless, recent scientific evidence suggests that the quantity of irreducible information measured during neuroimaging (Luppi et al., 2024) correlates with levels of arousal (from coma to waking states) and contents of consciousness (from eyes closed to watching a film).

Global Neuronal Workspace Theory

*Epistemological status: empirically grounded*

Global Neuronal Workspace Theory (GNWT) (Baars, 1993) argues that perception only becomes accessible to consciousness when it is widely broadcasted across the brain, i.e., within a “global workspace.” According to GNWT, unconscious processes remain localized and modular until information reaches a threshold for “ignition” and shared across the workspace (Mashour et al., 2020). GNWT emphasizes the role of recurrent, or re-entrant, long-rage loops between cortical areas for ignition to occur and the role of the prefrontal cortex and access consciousness, whereas IIT places importance on posterior regions and phenomenal consciousness. Critics of GNWT argue that the theory struggles to accommodate for the phenomenal character of subjective experience (Dalton, 1997). Recently, GNWT and IIT were tested through a neuroscientific adversarial collaboration that linked brain activity across imaging modalities to subjective contents of consciousness, finding evidence both in support and against each theory (Ferrante et al., 2025).

Recurrent Processing Theory

*Epistemological status: empirically grounded*

The Recurrent Processing Theory (RPT) arose from the neurobiological observation that re-entrant processing loops play a critical role in consciousness. RPT posits that conscious perception emerges specifically from local feedback loops between sensory cortical areas, rather than from widespread broadcasting. According to RPT, an initial feedforward sweep through early sensory areas enables basic processing, but it is the reentrant (feedback) activity of neuronal signals looping back into earlier sensory areas that produces conscious awareness. The visual masking paradigm disrupts this feedback but spares early feedforward signals, highlighting the necessity of recurrent loops for conscious perception (Lamme and Roelfsema, 2000). Neurophysiological evidence continues to support this conclusion. For example, conscious detection of stimuli has been shown to correspond to increased feedback connectivity between primary and secondary somatosensory cortices, whereas that feedback was absent during non-conscious perception (Auksztulewicz et al., 2012). While debates on whether local recurrent activity is sufficient for full conscious experience, RPT provides a mechanistically parsimonious account of how specific neural architectures could instantiate perceptual awareness.

Free-Energy Principle

*Epistemological status: empirically grounded*

The Free-Energy Principle (Friston, 2010; Friston et al., 2006) suggests that biological systems, including the brain, resist disorder by minimizing their “free energy,” the prediction errors (surprises), related to how the systems preserves itself. A system minimizes destructive surprises by updating predictions about the world and acting to gather more conforming sensory data. This framework is designed to offer a unifying account of theories of perception, action, and brain function (Parr et al., 2025), with some suggesting that this framework might also unify IIT and GNWT (Safron, 2020). More recently, authors behind the Free-Energy Principle have built on the existing framework while drawing parallels to Recurrent Processing Theory, in a new theory of consciousness called the Beautiful Loop Theory (Laukkonen et al., 2025).

Higher-Order Theories

*Epistemological status: empirically grounded*

Higher-Order Theories (HOTs) posit that a mental state becomes conscious when the system has a higher-order representation of that state. In other words, a perceptual state is conscious if the organism is aware of being in that state. Some neuroscientific models map these processes onto prefrontal cortex activity, suggesting that the availability of information for report is central to consciousness. However, HOTs are criticized for relying on introspective constructs and for lacking clear neural correlates across species or levels of consciousness (Lau and Rosenthal, 2011).

Quantum theories

*Epistemological status: speculative; contested as unfalsifiable*

Quantum theories propose that quantum mechanical phenomena, such as superposition, entanglement, and coherence, may play a role in the emergence of subjective experience. Several have been proposed, untestable using current technology, distinguishing them from the neuroscience-based theories discussed in this section. Among the most well-known, Orchestrated Objective Reduction theory (Hameroff and Penrose, 1998), posits that consciousness arises from quantum computations within neuronal microtubules. Others propose that consciousness is related to wave function collapse, a phenomenon where, until a quantum particle is observed, it exists simultaneously in multiple states, an interpretation that grants the mind an active role in generating the state of physical reality (Linked: Stapp, 1982). Others suggest that quantum theories may point to the mental and physical as expressions of a more fundamental reality, supporting the view of dual-aspect monism (Linked: Bohm, 2005). While proponents of quantum theories argue they might explain features of consciousness that resist classical explanation, these theories are controversial, from issues in quantum coherence surviving the environment of the brain (Linked: Tegmark, 2000), to challenges in falsifying them empirically.

Filter theories

*Epistemological status: speculative; contested as unfalsifiable*

Filter theories of consciousness begin with the metaphysical stance of idealism, that consciousness exists outside of brains. William James (1842–1910) described this as the “mother-sea” or “cosmic reservoir.” In this view, the brain merely acts a receiver, functioning to filter the vast ocean of consciousness to that which is functionally relevant. Today, filter theories of consciousness continue to hypothesize that the brain is not the generator of consciousness, but rather a neural filter (Kastrup, 2025; Kelly and Kelly, 2007), citing expanded states of consciousness, including near-death experiences, as evidence supporting this hypothesis (Woollacott and Weiler, 2025). Through a heterophenomenological or “attributional” approach, investigators may remain neutral as to the meaning attributed to these experiences while working to characterize them and uncover how individuals are left with such an impression (Taves, 2009).

While each theory provides a distinct account of consciousness, these theories are not equally concerned with the same explanatory problems. For Integrated Information Theory (IIT), there is an emphasis on addressing the hard problem of consciousness, which is to explain why any physical system should give rise to subjective experience at all. IIT’s solution is to propose that consciousness is identical to integrated information, specifically arising wherever systems possess recurrent causal structure, including brains, and non-brains alike, compatible with both idealist and physicalist interpretations. Global Neuronal Workspace Theory (GNWT) is primarily a theory of access consciousness, a physicalist account of what we are conscious of, explaining how information becomes globally available for report, but is less directly concerned with why that availability should feel like anything subjectively. Recurrent Processing Theory (RPT) similarly targets the neural mechanisms of perceptual awareness rather than explaining how subjective experience arises. Higher-Order Theories (HOTs) address what makes an organism aware of being in a conscious state, while the Free Energy Principle offers a unifying account of perception, action, and brain function, which includes consciousness. For the science of consciousness, these differences in the target of explanation determine what would count as evidence for or against each theory during hypothesis testing. However, not all theories are empirically falsifiable and it is important to draw contrasts between which theories are empirically grounded, and which remain in the domain of philosophical interpretations or speculative perspectives.

Each theory also generates a separate set of predictions. For IIT, GNWT, and RPT, there are competing predictions on the neuroanatomical locus of consciousness. IIT predicts that consciousness is primarily associated with posterior cortical areas where integrated information is highest, whereas GNWT predicts that conscious access requires ignition of a prefrontal-parietal global workspace. RPT predicts that local recurrent activity in sensory cortices is sufficient. The recent adversarial collaboration between IIT and GNWT found that neural markers of conscious content were most consistently located in posterior cortices during stimulus presentation, offering partial support for IIT and RPT (Ferrante et al., 2025). Competing predictions are also found with respect to phenomenal and access consciousness. IIT and RPT allow for rich phenomenal experience that is not accessible for report, motivating no-report paradigms, whereas GNWT and HOTs tie consciousness more closely to access and reportability. Together, the recent findings point toward a framework in which global integration, recurrent processing, and the structural correspondence between neural activity and phenomenal experience may play complementary roles.

In addition to those highlighted above, several other contemporary theories are not highlighted here (Seth and Bayne, 2022). While new theories emerge (Laukkonen et al., 2025), others call for synthesis. An ongoing challenge is to develop a model of consciousness that accounts for its variety across the domains of science, medicine, and practice (Kozuch, 2024). Clues to the future may come from the past. For Wundt, “internal perception” was a means to arrive at the structured elements of consciousness. Structuralism is now returning to prominence (Kleiner, 2024) as the structural properties of experience are theorized to correspond to structural properties of neural activity (Benjamin and Kob, 2023), thereby providing the scaffolding required to bridge the neurophenomenological divide.

### Opportunities for future investigation

There are numerous opportunities for future investigation in the science of consciousness. These directions are considered further in the medicine and practice sections below and summarized together in the Discussion. Here, we note that the recent adversarial collaboration between IIT and GNWT establishes a model for empirically testing opposing theoretical claims that should be extended to other theories that are empirically falsifiable. Quantum theories of consciousness, currently untestable with available technology, should be monitored as scientific advances may eventually bring some hypotheses within empirical reach. The neurophenomenological program initiated by Varela is a promising area that remains underdeveloped relative to its potential. Pairing multi-modal neuroimaging with micro-phenomenological approaches could meaningfully bridge the explanatory gap, especially as emerging neuroscientific methods now offer the means to reconstruct visual experience directly from brain activity through deep neural networks, transforming first-person experience into measurable visual outputs that can be empirically validated (Kamitani et al., 2025). These advanced neurophenomenological techniques will likely benefit the structural turn in consciousness science, which remains in need of empirical operationalization. If structural properties of experience correspond to structural properties of neural activity, experimental paradigms need to be designed that can test this correspondence directly. A critical and unanswered question that emerges is whether neurophenomenological correlations are truly causally bidirectional, that is, whether a neural state identified through recording is sufficient to artificially reproduce an experience through targeted stimulation. Closing this loop, by combining rigorous phenomenological assessment and decoding approaches with high-precision neurostimulation would represent one of the most significant advances in consciousness science.

## Consciousness in medicine

Medical perspectives, from neurological disorders to anesthesia, offer meaningful insights into healthy waking consciousness. Below, we highlight several cases from clinical settings. Here, consciousness is operationalized by level of arousal (wakefulness), and contents of consciousness.

### Neuroanatomical dysfunction

Several vignettes of neuroanatomical cases offer powerful insights into consciousness. In split-brain syndrome, the thick bundle of white matter tracks connecting the left and right hemispheres (the corpus callosum) is severed, often following surgical treatment for epilepsy. Here, visual stimuli presented to the right hemisphere (left visual field), now fail to reach language centers within the left hemisphere, resulting in a failure to name objects (Schechter and Bayne, 2021). Similarly, EEG finds neural oscillations failing to cross the hemispheric divide, underscoring the requirement of integrating otherwise modular brain signals for conscious processes (Avvenuti et al., 2020).

In spatial neglect, after a right hemisphere stroke, spatial processing is damaged as (Mesulam, 1999) patients neglect their left side entirely (Esposito et al., 2021), including the left-hand side of drawings. It often presents with anosognosia, the lack of awareness of a deficit. Right hemisphere strokes, including the one experienced by Jill Bolte Taylor, has been described as expansive, nirvana-like and an extension of a sense of self and awareness (Taylor, 2008).

Following a posterior traumatic brain injury, some “blind” patients nonetheless manage to walk around obstacles (de Gelder et al., 2008). This unconscious perception, termed blindsight (Aleci and Dutto, 2024), has been known for half a century (Weiskrantz et al., 1974). However, recent studies have revealed key neurobiological mechanisms, finding the brain works through alternate neural pathways in the visual system to maintain functionality (Kinoshita et al., 2019), underscoring the brain’s adaptive capacity and redundancy in functions, including for consciousness.

### Disorders of consciousness

**Figure 2:**
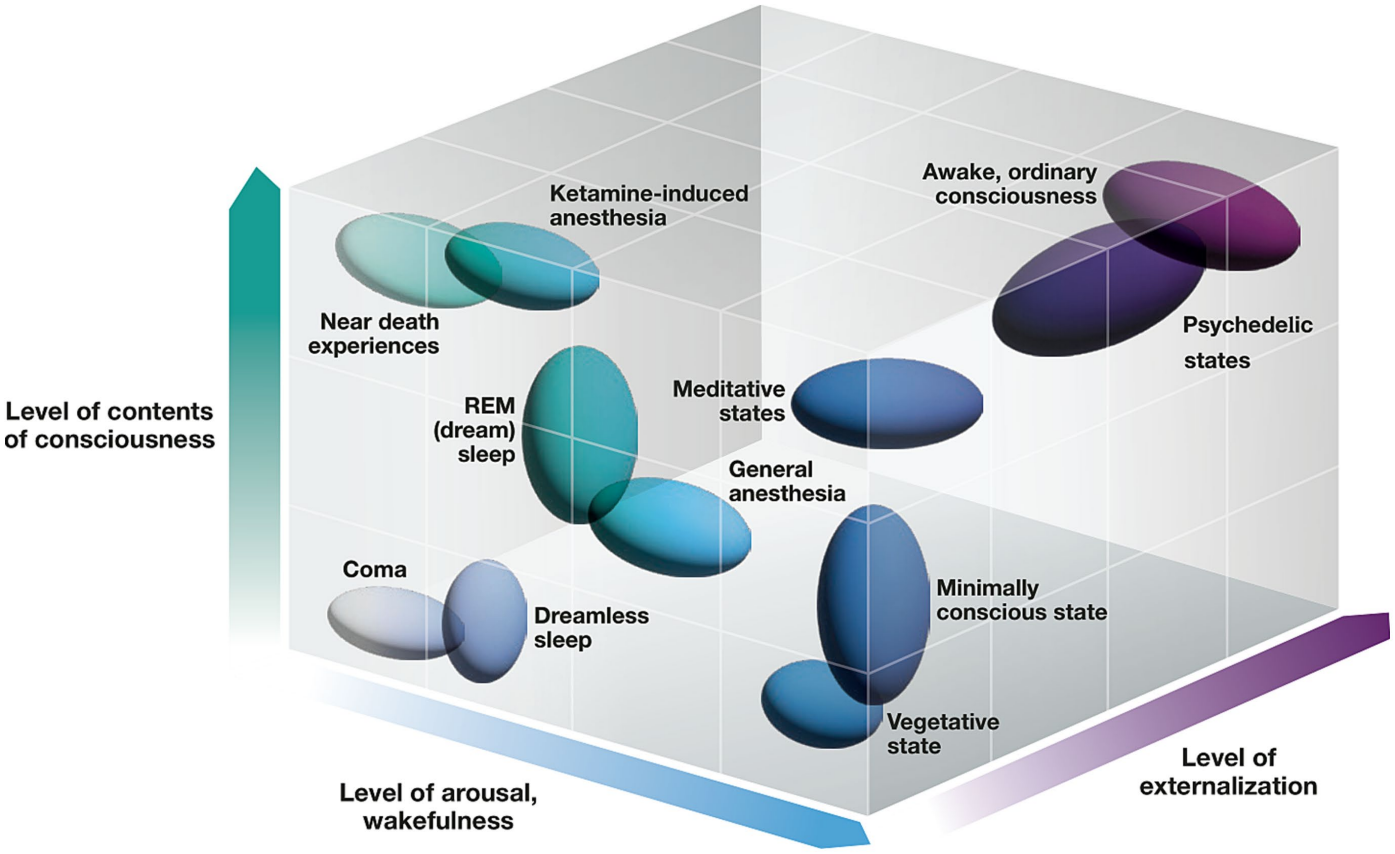
Disorders in the basic state space of consciousness. Here, we show major states and disorders of consciousness, indicating the level of arousal, contents, and externalization in healthy individuals and patients.

Disorders of consciousness are broad and multi-faceted (Figure 2). In the vegetative state, wakefulness occurs without responsiveness, yet evidence is emerging that patients can modulate brain activity to respond using thought alone (Monti et al., 2010; Owen et al., 2006). No single brain region is both necessary and sufficient for waking consciousness. While brainstem lesions typically cause immediate coma, disorders of consciousness can occur even when the brainstem is intact, indicating that the brainstem is not sufficient to sustain consciousness (Plum and Posner, 1982). In disorders of consciousness, neuroscientists have identified reduced activity in frontal and parietal cortices, informing NCCs (Koch et al., 2016) and corresponding to cortical disconnection syndromes (Laureys, 2005). EEG is suited for detecting brain activity that may be theorized to support conscious processes (Koch et al., 2016). High frequency oscillations, known as gamma oscillations, have been shown to correlate with attention. However, gamma synchrony can persist during anesthesia and dreamless sleep, ruling out gamma oscillations as an NCC (Murphy et al., 2011). The P300 waveform correlates with awareness during the attentional blink paradigm and detects covert consciousness in some vegetative patients (Faugeras et al., 2011). However, the P300 has also been described as a false positive (Silverstein et al., 2015), including in coma patients (Tzovara et al., 2015).

In 2013, a landmark EEG study identified a novel NCC by quantifying the neural complexity of brain responses following transcranial magnetic stimulation (TMS) (Casali et al., 2013). The study analyzed data, not only from patients with disorders of consciousness, but also from healthy subjects during wakefulness, dreaming, dreamless sleep, and anesthesia. The researchers reliably discriminated between levels of consciousness across conditions. Since that time, neural complexity has been explored across various complexity algorithms and has been described as a “common denominator of human consciousness,” with superior specificity for detecting consciousness over other measures of brain activity (Frohlich et al., 2022). Whereas neural complexity reduces in disorders of consciousness, neural complexity increases across the brain during a film or task (Mediano et al., 2021). Relatedly, both fMRI (Boly et al., 2015) and EEG (Mensen et al., 2018) studies report greater complexity when visual stimuli contained meaningful content, providing some empirical support for IIT. An increase in complexity across scalp electrodes is often not detected during auditory stimuli (Mediano et al., 2024), which can even reduce complexity across the brain (Bola et al., 2018; Orłowski and Bola, 2023). Only a small fraction of the cortex is dedicated to auditory processing (Pickles, 1988), whereas nearly half of the human cortex is involved in vision (Zeki, 1993), indicating that complexity may be linked to regional brain function.

### Neuromodulation

Various stimuli can modulate areas of the nervous system (Luan et al., 2014). These techniques are designed to ultimately optimize neural function. For consciousness, neuromodulation may restore or enhance aspects of conscious awareness. Recent studies using focused ultrasound have targeted brain regions in patients and healthy participants. In those with disorders of consciousness, after targeting the thalamus, behavioral responsiveness and recovery times improved (Cain et al., 2022). In healthy subjects, targeting the posterior cingulate cortex was also found to increase mindfulness and alter the sense of self and time (Lord et al., 2024). In another study, subcortical structures facilitated deep meditative states (Cain et al., 2024). With high spatial specificity and potential clinical applications, neuromodulation techniques are a promising future direction in consciousness research (Abellaneda-Pérez et al., 2024).

### Anesthesia

Anesthetics are essential for the study of consciousness (Mashour, 2024). General anesthesia includes anesthetic gases nitrous oxide and xenon, which block excitatory signaling at NMDA receptors across the brain to reduce excitatory neurotransmission (Colloc'h et al., 2007). Propofol, also used in general anesthesia, attenuates brain function by increasing inhibitory signaling in the brain via positive allosteric modulation of GABA receptors, the principal inhibitory receptor in the brain (Colloc'h et al., 2007). Downstream of these receptor mechanisms, anesthetics have been shown to induce a functional disconnection of the prefrontal cortex (John et al., 2001; Lee et al., 2013; Vlisides et al., 2017) an effect that is more profound during anesthesia relative to sleep (Zelmann et al., 2023) and described as providing support for GNWT (Demertzi et al., 2019; Huang et al., 2021). Furthermore, stimulation of prefrontal, but not parietal cortices, reversed the anesthetized state in rats (Pal et al., 2018). Preclinical studies have shown that various anesthetic agents with distinct molecular mechanisms uncouple or disintegrate the apical and basal compartments of cortical layer 5 pyramidal neurons, which in turn inhibit signal propagation within the thalamocortical system and suppress reentrant feedback connectivity from the prefrontal cortex (Aru et al., 2020; Suzuki and Larkum, 2020). Future studies should leverage anesthetics as a research tool to empirically test and differentiate theories of consciousness (Mashour, 2024).

### Near death experiences

Clinically related modifications of consciousness can also occur spontaneously, such as in the case of near-death experiences (NDEs). Despite arising spontaneously in uncontrolled settings, such as during accidents, the phenomenology of NDEs show remarkable similarity between one another (Martial et al., 2025). Following a major accident, the actor Jeremy Renner recently described what researchers have deemed “classic for near-death experiences,” specifically, that “There was no time, place or space, and nothing to see, except a kind of electric… inconceivable energy” (Astor, 2025). Descriptions of NDEs draw significant parallels to consciousness-modifying practices described below (Martial et al., 2025), underscoring the multiple realizability of various neural mechanisms for a particular experience (Alexander and Newell, 2017; Junger, 2024). “Collection of new data should be by far the highest priority until we really know something about what is going on in these unusual individuals and states.” (Kelly and Kelly, 2007).

### Opportunities for future investigation

Medical perspectives of consciousness point to several critical opportunities for future investigation. Neural complexity has emerged as the leading neural correlate of consciousness, demonstrating superiority over other forms of brain activity, including the integration of discrete brain networks, for mapping onto the level of conscious arousal and contents across various conditions. However, further validation from large datasets containing conditions across relevant axes of the state space of consciousness, including arousal and content, is needed to establish neural complexity as a unified, theory-neutral and full NCC. Neuromodulation by focused ultrasound is a promising tool for future investigation, including for uncaging and releasing anesthetics, such as propofol, to target specific brain regions for a high-resolution dissection of the neuroanatomy of consciousness (Wang et al., 2018). The neurobiology of NDEs also remains poorly understood, with data collection surrounding these medical cases, including structured micro-phenomenological assessments, representing a high-priority and tractable research opportunity.

## Consciousness in practice

In practice, consciousness is engaged in frameworks distinct from the scientific and medical domains above. Whereas science is primarily concerned with dissecting features of experience and neural correlates, and medicine is primarily concerned with resuscitating levels of arousal in disordered patients, practice encompasses the breadth and nuance of various contemplative and Indigenous traditions, including meditative and entheogenic practices that aim to cultivate and expand consciousness for ritual and community. This section emphasizes shifts to consciousness outside of normal waking states, shifts that inform the space and range that a mind can occupy, while uncovering underlying commonalities and continuity.

### Meditation

Meditative practices encompass a diversity of techniques, from open monitoring of the moment, to focused attention, such as on breathwork, or drumming (Fincham et al., 2023; Grof et al., 2008; Huels et al., 2021). The desired outcome is often a heightened awareness, including mindful awareness (Lutz et al., 2008), or non-dual awareness, where dualities of the self and other, or the mental and physical, are unified (Josipovic, 2014). Although research into meditation is limited by diversity in techniques and practitioners (Lippelt et al., 2014), these practices provide an essential comparator when examining the mind and brain effects of other consciousness-modifying techniques. In the brain during meditation, relative to the resting wakeful state or states of mind-wandering, a recent review found increases in neural complexity that were most pronounced in experienced meditators, potentially indicating higher levels of consciousness (Atad et al., 2025). EEG studies further report increased frontal theta and alpha power, representative of a relaxed yet alert state (Cahn and Polich, 2006), while fMRI studies report activity in brain regions associated with attention and interoception, including the anterior cingulate cortex and other frontal cortices (Boccia et al., 2015; Cahn and Polich, 2006). fMRI studies of network changes have also examined the inversely related default-mode network (DMN) central executive network (CEN). During focused attention on mantra, the inverse relationship between the networks was strengthened, whereas during non-dual awareness, this relationship was weakened, pointing to an attenuation of the duality of extrinsic and intrinsic experiences into a state of self-transcendence (Josipovic et al., 2012).

On the molecular level, several meditation studies have identified associations with the monoamine neurotransmitter systems, which include serotonin, melatonin, and the catecholamines dopamine, epinephrine, and norepinephrine (Britton et al., 2014; Jindal et al., 2013; Newberg and Iversen, 2003). Findings of increased serotonin levels support the notion that meditation induces an awakened state of “relaxed alertness” (Britton et al., 2014), while melatonin, which is synthesized downstream of serotonin, has also been shown to increase following meditation (Tooley et al., 2000). In a neuroimaging study of Yoga Nidra, a guided relaxation technique, dopamine tone increased at the dopamine 2 receptor and was associated with reduced desire for action (Kjaer et al., 2002). A separate study of an Isha Yoga retreat found that endogenous cannabinoid levels in the periphery increased by over 70% (Sadhasivam et al., 2020). Oxytocin, with known roles in social bonding, has also been studied during a single mindfulness session (Bellosta-Batalla et al., 2020) and after an 8-week program (Aygün et al., 2024), with both studies finding increased oxytocin levels, measured in saliva and plasma, respectively.

One underexplored question is whether elevating the concentrations of the brain’s endogenous signaling compounds that are observed during meditative states should facilitate or induce such a state, or conversely, whether blocking these systems, i.e., with a receptor antagonist, would interfere with its emergence. Using an oxytocin spray, one group of researchers showed that oxytocin increased positive emotions during meditation and increased spiritual beliefs, an effect that was moderated by genotypes related to oxytocin signaling (Van Cappellen et al., 2016). Together, meditative practices have begun to uncover the biological substrates of heightened conscious perception and awareness.

### Entheogens

“The feeling of being completely merged with the source of the universe can permanently change one’s theology and understanding of the cosmos.” For millennia, non-ordinary states of consciousness have been a central part of religious and spiritual practices. The quote above describes 5-MeO-DMT, a compound in plants used for thousands of years by Indigenous peoples of South America (Ermakova et al., 2022). These and other “entheogens,” meaning generating the divine from within, are described as teachers by Indigenous peoples (Kimmerer, 2013) and are also often classified by their activity at serotonin 5-HT2A receptors, as “psychedelic” compounds, meaning mind-manifesting (Huxley et al., 1999). Both names, “entheogen” or “psychedelic,” refer to a shared non-ordinary state of consciousness: “When the individual realizes experientially that the root of their being is synonymous with the root of all being, consciousness reaches – at least briefly – a cosmic proportion.” This realization has been described as the “Unitive Mystical Experience” (Sawyer, 2024) and a “metaphysical insight” often shared across non-ordinary states of consciousness (Jylkkä et al., 2025). Even if these experiences are difficult to measure, researchers point out that their beneficial effects are entirely quantifiable (Schneeberger, 2010).

How might science account for these experiences of powerful insight and observation (Taves, 2020)? The hypothesis that mind-manifesting compounds exists is largely untested (Hunt and Chefurka, 1976; Lindegaard, 2023). Some support has emerged from clinical trials finding that “psychological insight” induced by psychedelics predicts improved patient outcomes (Davis et al., 2021; Peill et al., 2022). More recently, an analysis of online anecdotal reports addressed whether individuals who consumed psychedelics reported similar novel observations about consciousness (Dikovskaya et al., 2025). Ketamine and salvia, which are not pharmacologically defined as psychedelics, were particularly associated with an immersive structural experience reported as a “wheel” or “vortex” often following reports of time dilation and self-dissolution. For example, “spinning furiously and gaining momentum down a vortex right into some grotesquely ironic inner dimension at the core of matter, time, space and thought.” Notably, a separate study found that ketamine and salvia reports were most similar to NDEs amongst hundreds of other pharmacological agents (Martial et al., 2019), lending validation to first-person ketamine and salvia reports as windows into reverse engineered or deconstructed states of consciousness, as opposed to mere drug-induced artifacts or hallucinations. Cannabis, acting through another distinct neural system, the endocannabinoid system, also shares in these structural phenomenological descriptions, with some reports indicating independent observations of moments unfolding in time in a pattern analogous to Hegel’s dialectic (Murray and Srinivasa-Desikan, 2022). Together, prior work informs entheogenic practices in occasioning mystical experiences (Griffiths et al., 2006) and revealing aspects of the nature of consciousness (Timmermann et al., 2025; Yaden et al., 2021), including latent metaphysical structures, or scaffoldings supporting consciousness.

**Figure 3:**
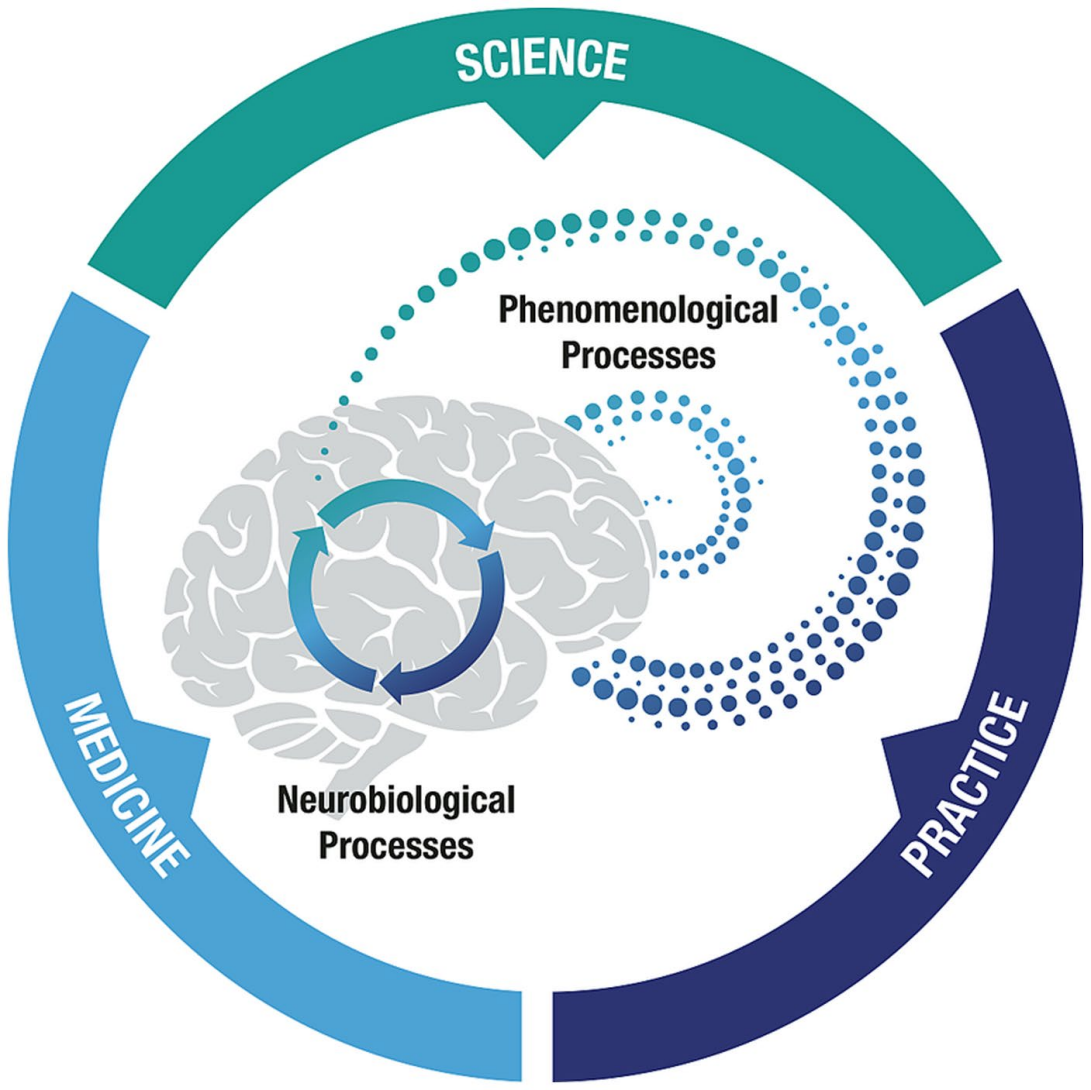
A neurophenomenological model of consciousness. Here, we show how three sections of the paper: Science, medicine, and practice, give rise to a new model wherein neurobiological processes (recurrent loops) and phenomenological processes (wheel/vortex structures) are set as cornerstones of the mind-brain architecture.

What is the neural basis for non-ordinary states of consciousness, from delusional hallucinations to insightful observations? Neuroimaging studies generally show an increase in the complexity of neural signals, and the connectivity of neural networks during non-ordinary states of consciousness (Carhart-Harris and Friston, 2019; Muthukumaraswamy et al., 2013; Timmermann et al., 2023). As the neural complexity and connectivity increases, brainwaves are often desynchronized. In particular, the alpha oscillation, which desynchronizes in response to brain activity, has been linked to ego dissolution (Muthukumaraswamy et al., 2013). Neural complexity might represent a marker of enhanced perception and observation, increasing even after low or “microdoses” of LSD (Murray et al., 2024), alongside self-reported mental clarity (Murray et al., 2021). The neural basis for deep metaphysical insight will require further research into such insights, observations, and neural correlates. Various neurobiological alterations, from the molecular to systems level, may give rise to similar phenomenological experiences, including meaningful insights. However, it is possible that the observation of deconstructed consciousness, or more specifically, vortex experiences during non-ordinary states, are specifically linked to the fundamental neurobiological architectures required for conscious awareness. Here, it is tempting to speculate that the spinning, wheel-like nature of phenomenological structure arises from an internal perception of the recursive processing engine of the brain itself, illuminating the bridge across the neurophenomenological divide (Figure 3).

### Consciousness movements

The emergence of a global “consciousness movement” has beginnings in the U. S. in the mid-20th century, with key events including San Francisco’s Human Be-In event preceding the 1967 “Summer of Love.” This event drew high profile speakers, including Timothy Leary, whose speech introduced the “turn on, tune in, drop out” slogan of the counterculture movement. This, and related events during the 1960s, served to catalyze a growing “human potential movement” that also drew inspiration from mid-century concepts and movements in psychology, including self-actualization and self-transcendence (Kripal, 2011). These trends, continuing today, reflect a growing societal inclination to understand and engage consciousness not only for therapeutic purposes but as a mode of self-discovery and transformation. These modern movements in consciousness draw from longstanding Indigenous, Tribal, and Eastern traditions. These once separate traditions are now finding common ground in a global consciousness movement, with Indigenous peoples of the Americas incorporating mindfulness meditation toward reversing generational stress and trauma (Clarke and Bird, 2020). Together, consciousness movements encourage practices that promote greater awareness while emphasizing the role that consciousness plays for individual wellbeing and human flourishing.

### Consciousness and technology

Technology is increasingly shaping the landscape of consciousness. The use of immersive virtual reality, brain-computer interfaces, and neurofeedback is expanding the possibilities for modulating and replicating awareness. Wearable devices can now monitor EEG signals providing real-time feedback. Increased integration between artificial intelligence, neuroimaging, and virtual reality may lay the groundwork for decoding neural correlates of conscious states, toward simulations and reproductions of conscious experiences. Farther into the future, might technologies allow for “uploading” one’s mind into an advanced artificial system, unlocking a transhumanist afterlife? (Huxley, 2015). Some have said “No, your digital avatar may seduce everyone into believing it is you without feeling anything” (Koch, 2024). Such issues evoke a deep sense of responsibility for the future of consciousness and technology, while underscoring current ethical issues related to consciousness.

### Opportunities for future investigation

Insights gained from the practice of consciousness point to further opportunities for investigation, work that is prominently advocated for by the Dalai Lama (Lama, 2002). The diverse engagement of distinct neural mechanisms across highly variable practices resulting in strikingly similar conscious experiences, including unitive mystical experiences, should not be framed as an inconvenient inconsistency, but rather an important intersection: what does this convergence tell us about the fundamental architecture of consciousness? The neural basis of structural experiences documented across ketamine, salvia, and other non-ordinary states requires dedicated investigation pairing phenomenological characterization with high-resolution neuroimaging to determine whether recursive phenomenology corresponds to the brain’s own recursive processing architecture. Finally, Indigenous and non-Western epistemological frameworks have not been integrated as genuine methodological strategies to mainstream research. Engaging these frameworks as sources of research hypotheses, rather than merely as cultural context, represents both a scientific opportunity and an equity imperative.

## Conclusion

Here, we have explored the domains of philosophy, science, medicine, and practice in hopes to encourage truly transdisciplinary testable research hypotheses that will have the potential to advance our understanding of consciousness and new aspects of consciousness we might discover. These pillars do not operate independently, but rather stand together to support an overall framework toward illuminating consciousness. Philosophy establishes a conceptual foundation, clarifying questions and answers. Science maps neural correlates and tests theoretical claims with increasing empirical precision. Medicine informs the conditions under which waking consciousness arises. Practice provides the range and depth to human experiences. From the earliest philosophical accounts to the latest scientific findings, persistent gaps in knowledge and consistent patterns and insights emerge. To close the explanatory gap between mind and brain, we recognize the importance of starting from experience itself as opposed to starting from physicalist accounts of brain structure and function. From this vantage point and across the domains, a recursive working model of consciousness comes to light, wherein phenomenological and neurobiological processes meet as cornerstones of a mind-brain architecture that unfolds over time. A continuous structure, uncovered through various forms of reverse engineering the mind and brain.

Critically, further investigation is required to adequately test this model and fill other major gaps in knowledge described in the sections above. From the science of consciousness, we recognize that the adversarial collaboration model established between IIT and GNWT should be extended to other falsifiable theories, including those related to the structure of consciousness that may have separable claims. Current neurophenomenological methods, now bolstered by the rapidly improving ability to reconstruct subjective visual phenomena with the aid of deep neural networks, offers a path toward empirically testing whether the structures of conscious experience correspond to structures of neural activity, and may ultimately unlock the ability to test whether brain states recorded during various experiences may be artificially induced to recreate them, addressing the nature of correlation and causation between brain and mind. From consciousness in medicine, it is clear that neural complexity has emerged as the leading candidate as a neural correlate of consciousness, welcoming future validation across large datasets spanning the full state space of consciousness, while focused ultrasound neuromodulation offers a high-resolution tool for dissecting the neuroanatomy of consciousness.

Together, these opportunities outline an integrated research program aimed at addressing the most fundamental unanswered questions about the nature of consciousness. *How is consciousness constructed?* The convergence of neurophenomenological methods, neural complexity measures, and adversarial theory testing offers a path toward empirically resolving whether consciousness arises from recurrent neural loops, integrated information, global broadcasting, or some combination thereof, and whether the structures of experience correspond to the structures of neural activity. *How can consciousness be measured and modified?* Neural complexity has emerged as the most promising measure of a unified conscious level, with neurophenomenology poised to map conscious contents and structure, while neuromodulation offers tools for deliberately altering conscious states with increasing precision. And *why does consciousness matter, not only for survival, but for human flourishing*? The contemplative and Indigenous traditions we highlight suggest that consciousness is not merely an epiphenomenon of biological computation, but a capacity that can be cultivated, expanded, and directed toward healing, insight, and connection. Together, we advocate for a science of experience, for exploration, and for healing, of ourselves and our world.
